# The Effects of Age at Weaning and Length of Lipid Supplementation on Growth, Metabolites, and Marbling of Young Steers

**DOI:** 10.3390/ani10101819

**Published:** 2020-10-06

**Authors:** Jessie E. Tipton, Linda K. Lewis, Ralph E. Ricks, Sebastian Maresca, Sebastian Lopez Valiente, Nathan M. Long

**Affiliations:** 1 Department of Animal and Veterinary Sciences, Clemson University, Clemson, SC 29634, USA; jetipto@g.clemson.edu (J.E.T.); lklewis@g.clemson.edu (L.K.L.); ericks@g.clemson.edu (R.E.R.); 2 Cuenca del Salado Experimental Station, National Institute of Agricultural Technology, Rauch, Buenos Aires, BA 7203, Argentina; maresca.sebastian@inta.gob.ar (S.M.); lopez.valiente@inta.gob.ar (S.L.V.)

**Keywords:** carcass quality, fat supplementation, fatty acid concentration, metabolites, weaning

## Abstract

**Simple Summary:**

Consumers value quality beef and producers are starting to look at the ways production decisions affect the long-term performance of the animals. Early weaning is a production option in many beef cattle production environments. We are looking at the addition of rumen by-pass lipids in addition to early weaning to increase the marbling of steers. The supplementation of rumen protected lipids’ increased plasma concentrations of fatty acids. Supplementation of rumen-protected lipids improved carcass quality of young steers by increasing marbling scores and lipid concentration of steaks without negatively impacting dressing percentage. Therefore, a combination of early weaning and rumen by-pass lipid supplementation can be used as management practices to meet current consumer demands.

**Abstract:**

The objective of this study was to determine how weaning age, days on supplements, and lipid supplementation affected the growth and marbling deposition of steers. Steers from a single sire were early weaned (n = 24) at 150 ± 11 days of age or traditionally weaned (n = 24) at 210 ± 11 days of age. Steers were assigned to control (n = 12/weaning group) or an isocaloric, isonitrogenous rumen by-pass lipid (RBL, n = 12/weaning group) for either 45 (n = 6/treatment) or 90 (n=6/treatment) days then harvested. Steer body weight (BW) was recorded on days −14 and −7, then BW and blood samples were collected on days 0, 22, 45, 66, and 90. The right rib section of each animal was collected for proximate analysis. *Longissimus dorsi* from RBL steers had increased lipids compared with control steers (3.6 ± 0.2 vs. 2.4 ± 0.2% on a wet basis; *p* < 0.0001). Steers fed for 90 days had greater (*p* = 0.02) concentrations of *Longissimus dorsi* lipid (3.3 ± 0.2%) than those fed for 45 days (2.7 ± 0.2%). There was a weaning age by treatment by days on feed interaction for intramuscular adipocyte diameter (*p* = 0.02) in which early weaned RBL fed for 90 days steers had an increased adipocyte diameter compared to the early weaned control fed for 90 and early weaned fed for 45 days steers with all other treatment groups as intermediates. Supplementation of RBL increased concentrations of C18:2, C20:4, and total fatty acids on days 45 and 90 (*p* ≤ 0.05). Data show that RBL supplementation increased the marbling content of the *Longissimus dorsi*. Furthermore, a longer period of supplementation resulted in increased adipose diameter.

## 1. Introduction

There is currently interest in improving the quality of beef carcasses by increasing the amount of marbling as well as the nutritional value of those fats by increasing the content of unsaturated fatty acids (USFAs; [1,2]). Increased marbling scores are associated with increased flavor, tenderness, and juiciness in a beef carcass [3]. Early weaning, removal of the calf from the dam prior to 180 days of age [4], has increased marbling content in beef carcasses compared with those weaned at the traditional 210 days of age because of the association of early weaning and concentrate feeding at a young age [5,6,7,8,9]. Supplementation of a rumen by-pass lipid (RBL) has also increased marbling in early weaned steers [10] and increased the amount of USFAs available for post-ruminal absorption by the small intestine [11]. Wang, et al. [12] showed that pathways responsible for regulating intramuscular adipose deposition are active as early as 7 months of age. Therefore, management practices used to increase marbling should be applied prior to or at this age.

Increased costs resulting from increased concentrate feeding associated with early weaning could deter producers from adopting early weaning. Therefore, identifying the optimal age to start supplementation as well as the optimal length of supplementation to yield the most improvement in the carcass is critical for the adoption of these practices. The objective of the present study was to determine if early weaned and the supplementation of a RBL for either 45 or 90 days post-weaning would increase carcass quality in steers prior to finishing. We hypothesized that early weaning would increase the amount of marbling in young steers and that the effects of RBL supplementation would increase the amount of USFAs and marbling content in young steers after only 45 days of supplementation.

## 2. Materials and Methods

All procedures were approved by Clemson University Animal Care and Use Committee (AUP #2016-029).

At 120 ± 11 days of age, all steers were vaccinated against *Pasteurella*, 5-way respiratory and *Leptospira*, and 7-way Clostridial bacteria (Presponse HM (intramuscular (IM)), Bovi-Shield Gold FP5 L5 (intramuscular (IM)), UtraChoice-7 (subcutaneous (SQ)), respectively; Pfizer, Exton, PA, USA). At 150 ± 11 days of age, body weight (BW) was recorded for 48 steers born to multiparous cows (3 to 11 years of age from 3 pasture groups) sired by a single Angus AI sire. All steers were castrated within 24 h of birth. Steers were sorted by dam’s breed type (Angus n = 19, Angus x Simmental n = 20, and Angus x Hereford n = 9) and BW, then randomly assigned by breed to one of two conventional weaning systems: early weaned (n = 24) or traditionally weaned (n = 24). Calves assigned the early weaning treatment were weaned from their dams at 150 ± 11 days of age and began the adjustment feeding period. Calves in the traditionally weaned treatment, returned to 2 pasture groups with their dams until weaning at 210 ± 11 days of age, when BW was collected, then the calves were removed from dams to begin the adjustment feeding period. Vaccinations were repeated at a time of abrupt weaning respective to the steers assigned weaning time (150 ± 11 days and 210 ± 11 days of age for early and traditionally weaned steers, respectively). At the time of weaning, steers were sorted into pairs according to breed type and BW to ensure one steer per RBL treatment in each pen (two steers per pen), then placed in 4.4 × 8.1 m pens for the duration of the study.

Throughout the study (adaption period and feeding trial), steers received ad libitum Bermudagrass hay (14.9% crude protein (CP), 2.3% ether extract (EE), 61.7% total digestible nutrients (TDN), 6.5 MJ/kg NEm, 32% acid detergent fiber (ADF), 66.3% neutral detergent fiber (NDF) on a dry matter (DM) basis). Hay was weighed and replenished daily at 06:30 h. Accumulated hay refusals throughout the adaption period and feeding trial, on days 0, 22, 45, 66, and 90 respective to the start of the trial, were weighed in order to calculate hay consumption per pen.

Over a period of two weeks after abrupt weaning, the calves were adjusted to a diet consisting of corn gluten feed (CGF; 91.6% DM, 21% CP, 87.2% TDN, 9.6 MJ/kg NEm, 5.2% EE on a DM basis) and oats (88% DM, 12.9% CP, 75.5% TDN, 8 MJ/kg NEm, 3.7% EE on a DM basis). During this period, CGF was added at 0.25 kg every 3.5 days to 2 kg of oats until reaching a final composition of 2 kg oats and 1 kg CGF. Immediately following the adaptation period (days −14 to −1), the steers were started on one of two rations for the feeding trial (day 0). Ration treatments were either a control (n = 12) ration consisting of 900 g CGF (n = 12) or a rumen by-pass lipid (RBL, n = 12) ration consisting of 143 g of Essentiom™ (Church and Dwight Co., Inc., Princeton, NJ, USA), 185 g of soybean meal (SBM), and 435 g of CGF. The fatty acid composition of Essentiom, CGF, and Bermudagrass hay are provided in Supplemental Table S1.

Steers per weaning treatment were also randomly allotted into groups that were fed for either 45 (n = 12/ weaning group) or 90 (n = 12/weaning group) days. Treatment groups were created to represent a 2 × 2 × 2 factorial design, made up of conventional weaning (early weaned vs. traditionally weaned), treatment ration (control vs. RBL), and the length of feeding trial in days (45 and 90 days). Treatment groups: early weaned steers fed a control diet for 45 days (EC45, n = 6), early weaned steers fed an RBL diet for 45 days (ERBL45, n = 6), early weaned steers fed a control diet for 90 days (EC90, n = 6), early weaned steers fed an RBL diet for 90 days (ERBL90, n = 6), traditionally weaned steers fed a control diet for 45 days (TC45, n = 6), traditionally weaned steers fed an RBL diet for 45 days (TRBL45, n = 6), traditionally weaned steers fed a control diet for 90 days (TC90, n = 6), and traditionally weaned steers fed an RBL diet for 90 days (TRBL90, n = 6). All rations were formulated and analyzed to be isonitrogenous and isocaloric (8.4 MJ NEm and 19.3 g CP on a dry matter basis). Steers were individually penned and fed treatment rations at 06:30 h daily. In addition, the steers were pen-fed 0.5 kg CGF and 1 kg oats per head at 19:00 h daily. On day 9 (relative to the start of treatment), 0.4 kg of CGF was added to both control and RBL rations in the mornings. Evening pen feed remained the same until day 68 when the ration was increased to 1 kg of CGF and 2 kg of oats per head.

Steer BW and blood samples were collected on days 0, 22, 45, 66, and 90, relative to the start of treatment. Two blood samples were taken at 06:00 h from each steer before feeding via jugular venipuncture. Blood samples were collected into 10 mL serum collection tubes (BD Vacutainer, Franklin Lakes, NJ, USA) and allowed to sit at room temperature for one hour before undergoing an overnight incubation at 4 °C. Following incubation, blood samples were centrifuged at 1800× *g* at 4 °C for 20 min and serum was poured off and stored at −20 °C until analysis. The second blood sample was also collected from each steer into 10 mL tubes containing EDTA (Covidien Monoject™, Mansfield, MA, USA) and immediately put on ice. Within 1 h of collection, the samples were centrifuged at 1800× *g* at 4 °C for 20 min. Plasma was then decanted and stored at −20 °C until analysis.

At the end of treatment (either 45 or 90 days after the initiation of treatment), steers body weight was recorded and then steers were transported 128.8 km to a commercial abattoir for slaughter after a 12 h fasting period. Age of the animals at the time of slaughter were as follows: EC45 and ERBL45 were 209 ± 11 days of age, EC90 and ERBL90 were 254 ± 11 days of age, TC45 and TRBL45 were 269 ± 11 days of age, and TC90 and TRBL90 were 314 ± 11 days of age. After slaughter, hot carcass weight (HCW) was recorded. Dressing percentage was calculated as HCW/body weight prior to shipping for harvest. Before carcasses were moved to the freezer, adipose samples were taken from the subcutaneous (SQ), perirenal (PR), and intramuscular (IM) depots within 35 min from exsanguination. Two samples were immediately placed in 10% neutral buffered formalin and eventually embedded in paraffin for histology. Another sample from each adipose depot was snap frozen for biochemical analyses. After a 24 h chill, the right rib section of each carcass was collected, put on ice, and transported to Clemson University. The same trained professional evaluated each rib section for ribeye area (REA) and marbling score in accordance with the USDA standards [13]. In addition, two steaks were cut from each section and all external fat was removed. Two intramuscular adipose samples were collected from one of these two steaks and then immediately placed in 10% neutral buffered formalin and later embedded in paraffin for histology. The remaining steak was vacuum packaged and stored at −20 °C for proximate analysis.

### 2.1. Proximate Analysis of Steaks

One steak per steer was allowed to partially thaw, and then was cubed and pulverized in a Blixer 3 Series D (Rovot Coupe USA. Inc., Ridgeland, MS, USA). Samples were then packaged and stored at −20 °C until analysis. Percent moisture and lipid content of each sample was determined in duplicate according to Method 950.46 and Method 960.39 [14]. Total lipid extraction was evaluated by washing samples with petroleum ether (EMD Millipore Corporation, Billerica, MA, USA) in a Soxhlet extract apparatus for approximately 24 h. Intra-assay coefficient of variation (CV) for the percentage of moisture and percent total lipid (ether extract) were 4.5% and 4.8%, respectively.

### 2.2. Biochemical Assays

All samples were assayed in triplicate. Plasma triglyceride and cholesterol concentrations were determined using previously validated colorimetric assays (Pointe Scientific, Inc., Canton, MI, USA; [15]). The intra- and inter-assay CV for triglyceride concentrations were 4.1% and 3.9%, respectively. Cholesterol concentrations had an intra-assay CV of 3.1% and an inter-assay of 3.8%. Using previously published procedures [16], plasma glucose concentrations were measured colorimetrically (Liquid Glucose Hexokinase, Reagent, Pointe Scientific, Inc., Canton, MI, USA) with a 2.9% inter-assay CV and a 3.1% intra-assay CV. Insulin samples were measured in duplicate in a singular assay by Coat-A-Count insulin RIA (Siemens Medical Solutions Diagnostics, Los Angeles, CA, USA) with an intra-assay CV of 3.7% and sensitivity of 0.2 ng/mL. Insulin assay has been validated in our lab previously [16].

### 2.3. Fatty Acid Analysis

Duplicate 1 mL serum aliquots from all steers were lyophilized (HarvestRight, North Salt Lake, UT, USA) and then transmethylated according to Park and Goins [17]. An internal standard, methyl tricosanoic (C23:0) was incorporated into each sample during methylation. Each sample of fatty acid methyl esters was analyzed using a Shimadzu GC-2014 gas chromatograph equipped with a Shimadzu AOC-20s automatic sampler. Separations were completed using a 60 m high resolution gas chromatography column (Agilent Technologies, Inc., Santa Clara, CA, USA). Samples were run at a split ratio of 10:1. Fatty acids were identified by comparing the retention times of known standards.

### 2.4. Histological Analysis

All SQ, PR, and IM adipose samples were fixed in 10% neutral buffered formalin, embedded in paraffin and sectioned at 10 µm using a Leica RM 2165 microtome (Meyer Instruments, INC., Houston, TX, USA). Two cross sections of adipose taken 5 sections apart were placed on slides (3 slides per sample). Sections were stained with hematoxlyin and eosin Y then evaluated with a Meiji MT3410L biological microscope (New York Microscope Company, Inc., Hicksville, NY, USA). Images were recorded at 20× magnification using Vixia HFS30 digital camera (Canon, Tokyo, Japan). Fields were chosen so that the field was composed mostly of regularly shaped adipose cells. Both SQ and PR adipocyte area was determined in 12 fields per animal using Image J software (National Institutes of Health, Bethesda, MD, USA). Adipocyte area was averaged across the 12 fields for an average adipocyte area per animal. Intramuscular adipocyte area was determined using 6 fields per animal with Image J software. Adipocyte area was averaged across the 6 fields for an average adipocyte area per animal. These procedures have previously been validated by Long et al. [18].

### 2.5. Statistical Analysis

Hay intake was collected as a pen (two steers per pen) while all other characteristics were collected based on the ration (individually supplemented). Thus, individual steer was used as the experimental unit for performance data, carcass measurements, proximate analysis, and metabolites while the pen was the experimental unit for hay intake. Hay intake during the feeding trial was analyzed using PROC MIXED (SAS Inst. Inc., Cary, NC, USA) with the weaning group, days on feed, and their interaction as fixed effects in the model statement. Birthweight as well as all BW and change in BW throughout the experiment, HCW, dressing percent, REA, marbling score, dry matter, ether extract, adipocyte diameters, fatty acid concentrations on days 0, 45, and 90 were analyzed using the MIXED model with the weaning group, days on feed, and treatment in the model as fixed effects and any interactions were left in the model statement that had a *p* < 0.1. Concentrations of plasma glucose, insulin, glucose to insulin ratio, triglycerides, and cholesterol were analyzed as repeated measures using the MIXED model of SAS with the weaning group, days on feed, treatment, and any interactions were left in the model statement that had a *p* < 0.1. Maternal breed type was initially included in all models as a random variable but found to be non-significant (*p* > 0.39) and therefore excluded from the final model. The P-diff option of SAS was used for the separation of means after a significant *p* value was determined. Data are presented as least square means ± SEM. Data are considered significant at *p* < 0.05 and a tendency is indicated at 0.05 ≤ *p* ≥ 0.10.

## 3. Results

### 3.1. Animal Performance

Steer BW from birth through the end of the treatment and change in BW during treatment are provided in Table 1. At every time point after weaning, traditionally weaned steers had an increased BW compared with early weaned steers (*p* < 0.0001). Treatment did not affect BW (*p* ≥ 0.16). During adaptation, traditionally weaned steers consumed more hay than early weaned steers (40.8 ± 0.8 vs. 24.8 ± 0.8 kg, respectively, *p* < 0.0001). There was a treatment* day effect (*p* = 0.0001) in which traditionally weaned steers fed for 45 d consumed more hay than early weaned steers fed for 45 d while on treatment (207.8 ± 7.1, 156.0 ± 7.1 kg, respectively), and similarly, traditionally weaned steers fed for 90 d consumed more hay than early weaned steers fed for 90 d while on treatment (424.9 ± 7.1 and 312.3 ± 7.1 kg, respectively).

### 3.2. Proximate Analysis of Steaks

Carcass measurements and steak proximate analysis data are presented in Table 2. Steers in the traditionally weaned group had a greater HCW than steers in the early weaned group (*p* < 0.001). Steers fed for 90 days also had a greater HCW and dressing percentage than those fed for 45 days (*p* < 0.0001 and *p* = 0.01). Supplementation with RBL had no effect of HCW at either harvest time. Ribeye area was not affected by the weaning group, treatment, or days on feed (*p* ≥ 0.19). Marbling score was increased in steers in the RBL treatment (*p* < 0.0001) compared with those in the control treatment as well as in steers fed for 90 days compared with steers fed for 45 days (*p* < 0.0001 and *p* = 0.0002, respectively). A treatment by day interaction (*p* = 0.02) for DM in which a greater concentration of DM in steaks from control steers fed for 90 days was observed than those fed for 45 days. Conversely, steaks from RBL steers fed for 45 days had a greater DM compared to RBL steers fed for 90 days (*p* = 0.02). Steaks from RBL steers had a greater percent of lipid than steaks from control steers (*p* < 0.0001). In addition, steaks from steers fed for 90 days had greater lipid concentration than steaks from steers fed for 45 days (*p* = 0.02).

### 3.3. Histological Analysis

**Figure 1:**
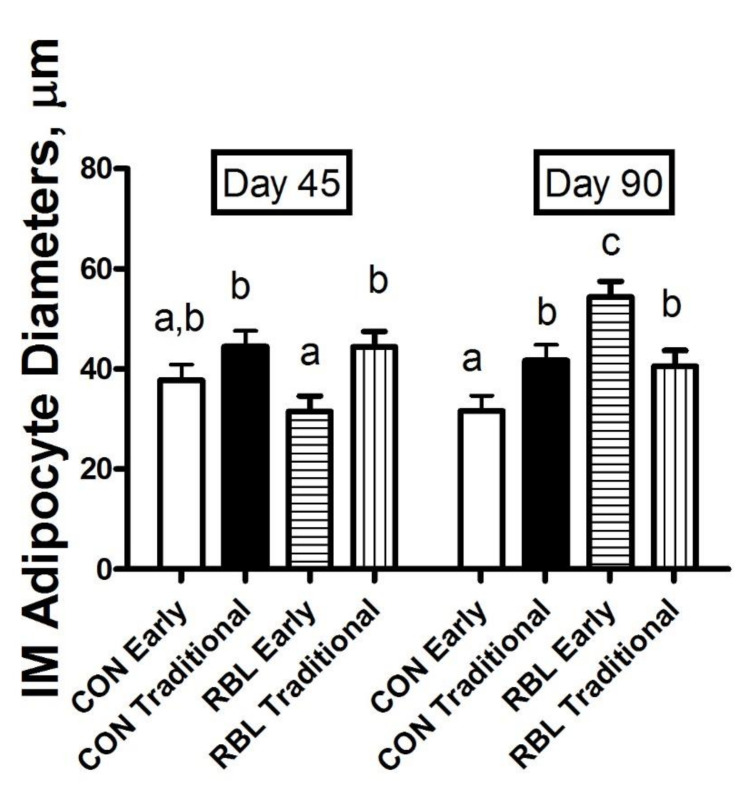
Intramuscualar (IM) adipocyte diameters (µm) of steers individually supplemented an isocaloric, isonitrogenous diet with no rumen by-pass lipid (CON; n = 24) or with a rumen by-pass lipid (Essentiom™; RBL; n = 24) daily and fed for a duration of either 45 or 90 d. Values are ± SEM. a, b, c: Means without common letters indicate significant differences (weaning × treatment × day: *p* = 0.02).

Adipocyte diameter for SQ or PR depots was not influenced by age at weaning or lipid supplementation or their interactions (*p* < 0.31). Steers fed for 90 days had an average SQ adipocyte diameter of 109.3 ± 7.0 µm, whereas those fed for 45 days had an average of 87.1 ± 7.2 µm (*p* = 0.03). Similarly, in the PR depot, steers fed for 90 days had an average adipocyte diameter of 140.14 ± 5.6 µm and steers fed for 45 days had an average diameter of 111.2 ± 6 µm (*p* = 0.001). Intramuscular adipocyte diameters are shown in Figure 1. In the IM depot, there was a three-way interaction between the weaning group, treatment, and days on feed in which ERBL90 steers have an increased adipocyte diameter compared to EC90 and ERBL45 steers with EC45, TC90, TC45, TRBL90, and TRBL45 steers as intermediates (*p* = 0.02).

### 3.4. Biochemical Assay

**Figure 2:**
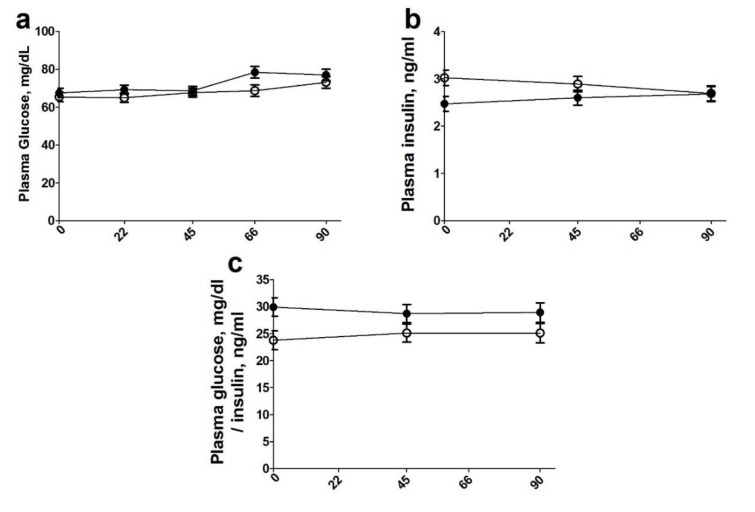
Plasma glucose (**a**) and insulin (**b**) concentrations as well as glucose to insulin ratios (**c**) on d 0, 22, 45, 66, and 90 (*y* axis) of the treatment for steers individually supplemented an isocaloric, isonitrogenous diet with no rumen by-pass lipid (CON; n = 24; open circle) or with a rumen by-pass lipid (Essentiom™; RBL; n = 24; closed circle) daily. Values are presented as ± SEM (glucose treatment: *p* = 0.03; day: *p* = 0.004. Insulin treatment: *p* = 0.02. Glucose to insulin ratio: treatment: *p* = 0.03).

**Figure 3:**
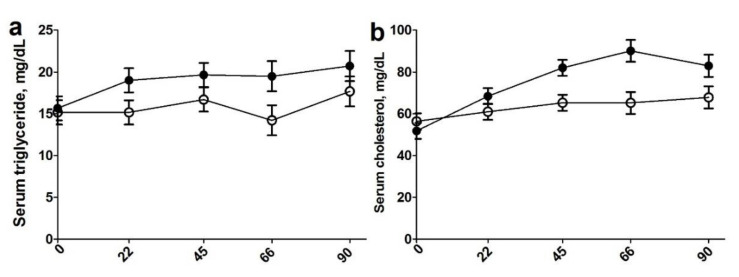
Plasma triglycerides (**a**) and cholesterol (**b**) concentrations on d 0, 22, 45, 66, and 90 (*y* axis) of treatment for steers individually supplemented an isocaloric, isonitrogenous diet with no rumen by-pass lipid (CON; n = 24; open circle) or with a rumen by-pass lipid (Essentiom™; RBL; n = 24; closed circle) daily. Values are ± SEM (Treatment: *p* = 0.002; Day: *p* = 0.03 and Treatment × Day *p* = 0.01, respectively).

As shown in Figure 2a, there was a treatment effect wherein steers fed an RBL diet had increased (*p* = 0.03) circulating glucose compared with control-fed steers. Steers fed control diets had increased circulating insulin compared with those fed RBL diets (Figure 2b; *p* = 0.02). This led to RBL steers having an increased glucose to insulin ratio (Figure 2c; *p* = 0.03). In addition, there was an effect of a day of treatment where increased plasma glucose concentrations on days 66 and 90 were observed when compared with days 0, 22, and 45 (*p* = 0.004). Traditionally weaned steers also had a tendency for increased circulating insulin concentrations compared with early weaned steers (*p* = 0.07). Plasma triglyceride (Figure 3a) concentrations were increased in RBL-fed steers compared with control-fed steers (*p* = 0.002), as well as in traditionally weaned compared to early weaned steers (*p* = 0.03). An effect of day of treatment was observed with an increase in triglyceride concentrations on day 90 compared with day 0 with days 22–66 as an intermediate (*p* < 0.03). Plasma cholesterol concentrations (Figure 3b) were increased (*p* = 0.01) in RBL-fed steers over control fed steers starting at day 22 through the end of treatment.

### 3.5. Fatty Acid Content

There was a significant day effect (*p* < 0.001) for both total and individual fatty acids but no day interactions with other variables so serum FA content is presented by day. Specific and total serum FA concentrations on day d 0 are given in Table 3. There were increased concentrations of C14:0, C20:0, and C18:3 in traditionally weaned compared to early weaned steers (*p* ≤ 0.04). Concentrations of C18:1 cis 9, C18:2, were increased in early weaned steers compared to traditionally weaned steers on day 0 (*p* ≤ 0.05). There was a tendency for control steers to start treatment with increased concentrations of C14:0 and C18:3 compared to RBL steers (*p* = 0.10).

Concentrations of specific and total serum FAs on day 45 are shown in Table 4. Steers in the RBL treatment had an increase in concentrations of C12:0, C14:0, C16:0, C18:0, C18:2, C20:0, C20:4, and total FA on day 45 when compared to control steers (*p* ≤ 0.05). Concentrations of C16:1 were increased in control steers over RBL steers on day 45 (*p* < 0.0001). Early weaned steers had an increase in C16:1 and C20:0 on d 45 over traditionally weaned steers (*p* < 0.0001) on day 45. A treatment by age at weaning interaction (*p* = 0.03) for 18:1 cis 9 concentrations was observed in which early weaned RBL steers were increased compared with traditionally weaned control and traditionally weaned RBL, while early weaned control served as an intermediate. There were no differences (*p* > 0.05) observed for C18:1 trans 9 or C18:3 at day 45 due to treatment or age at weaning.

Specific and total serum FA concentrations on day 90 are shown in Table 5. Traditionally weaned steers had increased concentrations of C12:0, C14:0, C16:0, C16:1, C18:0, C18:1 trans 9, C18:2, C18:3, C20:4, and total FAs on day 90 compared with early weaned steers (*p* ≤ 0.008). There was a tendency for traditionally weaned calves to have increased concentrations of C20:0 on d 90 when compared with early weaned calves (*p* = 0.06). Concentrations of C16:0, C18:2, C20:4, and total FAs were increased in RBL calves over control calves on day 90 (*p* ≤ 0.008). Control-fed steers had increased concentrations of C16:1 and C18:1 trans 9 when compared with RBL steers on day 90 (*p* ≤ 0.004). There was a treatment by weaning group interaction for C18:1 cis 9 in which early weaned control calves had decreased concentrations compared with early weaned RBL and traditionally weaned control calves with traditionally weaned RBL calves as an intermediate (*p* = 0.01).

## 4. Discussion

Our lab has previously shown that early weaned steers fed an unsaturated RBL supplement during the growing phase and finished in a feedlot had increased marbling scores compared with those fed a control diet during the growing phase [10]. However, there is difficulty discerning between the effects of calf age and supplementation on growth and carcass characteristics. To the authors’ knowledge, this is the first study to show how age at weaning, days on feed, and the supplementation of a RBL affects BW gain, glucose and insulin concentrations, circulating serum FA concentrations, and adipose deposition in young steers at the end of the supplementation period before being finished.

Since calves were blocked to treatments by initial BW, and there was a lack of difference in BW or change in BW between the control and RBL treatment, this demonstrates that the diets were isocaloric and isonitrogenous. This is important because it shows that the effects reported are due to the RBL treatment and not an energy effect. Many ruminant fat supplementation studies with isocaloric and isonitrogenous diets also show no differences in BW related to treatment [19,20,21]. In the current study, traditionally weaned steers had increased BW at all time periods post-weaning. The fact that early weaned steers tend to be lighter are supported by previous research that showed that steers weaned at 89 days of age and grazed pastures with supplement provided at 1% of BW were lighter at all time periods post-weaning [22]. Steers that were weaned at 74 days of age and placed on rye grass or fed concentrate for 90 days then placed on concentrate were of similar weight to traditionally weaned steers at 180 days of age [6]. However, when calves in early weaned systems are fed high concentrate diets they have increased BW at the time of normal-weaning compared with those left with their dams and continued to have increased BW at entry into the feedlot [6,8,23]. In these studies, calves were of similar age at each weight point. In the present study, the traditionally weaned group was approximately 60 days of age older than the early weaned group at each weigh date.

The increased hay intake between traditionally weaned steers and early weaned steers fed for the same number of days is similar to other early weaned studies that show decreased dry matter intake in early weaned steers compared with traditionally weaned steers during the growing period [7,23]. In a finishing study, daily DMI was decreased in early weaned calves compared with traditionally weaned steers, but these calves also experienced more days in the feedlot, which eliminated differences in total DMI between the weaning groups [9]. In addition to reduced DMI, early weaned steers are typically more efficient than traditionally weaned steers as demonstrated by improved gain:feed ratios and average daily gain [7,23].

The lack of HCW differences between the control- and RBL-treated steers again shows that the treatments were isocaloric. In the current study, traditionally weaned steers had increased HCW compared with early weaned steers. Similarly, when steers weaned at 100 days of age were harvested at similar BF thickness, they had decreased HCW compared with steers weaned at 200 days of age [23]. The authors attributed this difference to high concentrate feeding in the early weaned calves and thus encouraging fat accumulation at lighter weights. There are finishing studies that show HCW is increased in early weaned steers compared with traditionally weaned steers when cattle are finished to common BF thickness or common BW [5,8]. The increase in HCW in steers fed for 90 days compared to steers fed for 45 days in the present study is consistent with other studies that show a linear increase in HCW with increasing days of high concentrate feeding [24,25,26]. The increased days of age in the traditionally weaned group as well as calves fed for 90 days could also account for the increased HCW. A serial slaughter study shows that increased days of age resulted in increased HCW [27].

Increased marbling scores were observed in RBL steers regardless of the length of supplementation or weaning age. It must be noted that these steers in this study were 280 to 320 kg live weight at harvest and were at most 314 + 11 days of age, they were not finished steers. This resulted in the reduced marbling scores compared to finished steers in other published papers. Steers fed a RBL during the growing phase and then finished in a feedlot had increased marbling scores compared with those fed a control diet during the growing phase and would have resulted in greater carcasses values [10]. Steers weaned at 7 months of age and supplemented with a protected fat source for a 28 days pre-conditioning period produced carcasses with increased marbling scores compared to those not supplemented [28]. Increases in marbling scores as days on feed increase are also consistent with other studies that show either a linear or a curvilinear increase with increasing days on high concentrate feed [24,25,26]. The increase in the fat content of the longissimus dorsi is further demonstrated by the decrease in DM seen in steers fed for 90 days in the present study. USDA quality grades are assigned based on the amount of marbling present in the Longissimus muscle between the 12th and 13th ribs of a beef carcass [29]. Increasing marbling scores is a particular interest in cattle because it increases palatability, flavor, and juiciness [3]. Because of the increased consumer appeal with carcasses of higher marbling content, producers can be given premiums or deductions based on the quality grade that their carcasses are assigned [30,31].

Adipocytes grow both by hyperplasia and hypertrophy [32]. While it has been thought that intramuscular fat is a later maturing adipose depot [32,33], gene expression associated with hyperplasia, the increase in cell number, is detectable as early as 7 months of age [12]. Our results show that after only 45 days of supplementation in early weaned steers (harvested at 209 + 11 days of age), there are increases in the hyperplasia of adipocytes in the IMF depot, evidenced by decreased adipocyte diameter. However, after 90 days of supplementation, these differences are eliminated; suggesting that lengthy supplementation could result in the early maturation of IMF adipocytes. We also show that RBL supplementation did not affect hyperplasia in traditionally weaned steers. Steers weaned at 150 days of age and then supplemented a RBL showed a decrease in the diameter of IMF adipocytes compared to those fed isocaloric control diets after finishing, indicating an increase in adipocyte hyperplasia [10]. As animals age, terminally differentiated cells fill with lipid, causing the adipocyte to expand and become more rounded [32]. The expansion of adipocyte cells occurs as excess energy is stored in the form of triacylglycerols in the process known as lipogenesis [34,35]. In agreement with this, increased SQ and PR adipocyte diameter was observed in steers fed for 90 days over steers fed for 45 days in the present study.

Serum glucose, triglycerides, and cholesterol were increased in RBL steers. This is consistent with previous findings in our lab showing that RBL supplementation increased triglyceride and cholesterol concentrations after 55 days of supplementation [10]. A combination of increased triglyceride and cholesterol content was displayed in heifers receiving supplementations of calcium salts of fatty acids 5 days/week [15,36]. These increased triglycerides are probable due to the over 300% increased amount of dietary lipids that the RBL steers are consuming in the supplement compared to control steers (143.4 vs. 46.8 g) Increasing triglycerides in conjunction with increasing total FA concentration in RBL could result from increasing amounts of polyunsaturated fatty acids (PUFA) available for post-ruminal absorption. Increased cholesterol concentrations were observed in heifers supplemented with whole sunflower seeds starting at 4 months of age [21]. Rumen by-pass lipid supplementation increased serum concentrations of cholesterol and glucose in ewes during the breeding season [36,37]. The increased glucose to insulin ratio in RBL steers in our study is indicative of the increasing insulin sensitivity, which is commonly shown in fat supplementation studies. Cartiff, Fellner, and Eisemann [38] showed that the supplementation of fish oils with calcium salt increased insulin sensitivity in growing steers compared to the steers fed diets supplemented with saturated fatty acids.

Traditionally, USFAs are only available for absorption in the small intestine in relatively small amounts because of biohydrogenation in the rumen which causes them to become saturated [11]. Almost all fatty acids elevated in the RBL steers are the ones supplied in the RBL lipid source. The one fatty acid elevated in RBL steers not found in Essentiom is C20:4. Other research has found that when you supplement rumen by-pass lipids that are high in unsaturated C18 fatty acids, this leads to an increased serum C20:4 concentration (10,15). This is probably due to increased unsaturated C18 fatty acids available for conversion to C20:4. The relatively high levels of SFAs traditionally associated with beef products in conjunction with the negative effects of SFA consumption such as increased cholesterol and hypertension has caused a public desire for beef products with increased USFA content [1,2,30]. The use of calcium salts has been shown to slow this process and make USFAs more available for incorporation into beef products [11]. Increasing the content of USFAs in beef products may benefit meat animals as fatty acids have regulatory properties on adipocyte development [34], which may influence the meat quality and economic value of the meat. In the present study, we found increased concentrations of total fatty acids and increased PUFA concentrations in RBL supplemented steers after 45 and 90 d of supplementation. Consistent with our findings, Cooke et al. [28] showed that fat supplementation increased total FA and PUFA concentrations in the plasma of forage-fed beef cattle. Similar to Mangrum et al. [10], RBL supplementation high in linoleic concentration resulted in increased linoleic content on days 45 and 90. Increasing total and specific fatty acids were seen in early gestation heifers and lactating cows supplemented with RBL for 3 weeks Cook et al. [39]. However, frequency of an equivalent amount of supplementation (3, 5, or 7 days/week) showed minor to no differences in the fatty acid profile of these animals [39]. However, Long and others [15] showed that the RBL supplementation of heifers 5 days/week increased the total and specific serum fatty acid concentrations.

## 5. Conclusions

In conclusion, early weaned steers produced a lighter weight carcass of similar quality to traditionally weaned steers. Supplementation of RBL improved carcass quality of young steers by increasing marbling scores and the lipid concentration of steaks without negatively impacting dressing percentage. Furthermore, RBL supplementation increased insulin sensitivity, glucose, triglycerides, cholesterol, and serum-specific and total fatty acid content. Steers starting supplementation at 150 days of age respond to lipid supplementation in a similar manner to those starting supplementation at 210 days of age. A longer supplementation period increases fat content and carcass weight in young steers. Therefore, a combination of early weaned and RBL supplementation can be used as management practices to meet current consumer demands. Further studies aimed to correlate the FA profile of serum with that of meat are needed to better respond to consumer demand for healthy food.

## Figures and Tables

**Table 1:** Body weight (BW) and change in BW of young steers weaned at different ages ^1^ and supplemented a control (CON) or rumen by-pass (RBL) treatment.

Age at BW Collection	Weaning Group		Treatment		*p*-Value	
	Early ^1^	Traditional ^1^	CON	RBL	Weaning	Treatment
n ^2^	24/12	24/12	24/12	24/12	-	-
BW, Kg						
Birth Weight	35.2 ± 1.2	33.9 ± 1.1	33.8 ± 1.1	35.3 ± 1.2	0.66	0.84
150 d of age	195.9 ± 4.8	195.6 ± 4.6	195.0 ± 4.7	196.5 ± 4.6	0.97	0.81
d-14 ^3^	195.9 ± 4.8	240.5 ± 5.3	218.6 ± 5.5	217.72 ± 5.3	<0.0001	0.90
d-7	196.4 ± 4.5	240.1 ± 4.2	218.6 ± 4.4	217.9 ± 4.2	<0.0001	0.90
d 0 ^4^	207.4 ± 4.9	246.4 ± 4.7	228.2 ± 4.8	225.6 ± 4.7	<0.0001	0.69
d 22	231.1 ± 4.6	269.1 ± 4.4	252.3 ± 4.5	247.9 ± 4.4	<0.0001	0.49
d 45	252.7 ± 4.8	288.8 ± 4.5	272.6 ± 4.7	268.9 ± 4.5	<0.0001	0.58
d 66	262.9 ± 4.7	313.9 ± 4.6	293.4 ± 4.7	283.4 ± 4.6	<0.0001	0.16
d 90	284.2 ± 5.4	322.5 ± 5.2	306.6 ± 5.3	300.1 ± 5.3	<0.0001	0.40
Change in BW, Kg						
Adaptation ^3^	11.6 ± 3.3	5.9 ± 3.1	9.6 ± 3.2	7.8 ± 3.1	0.21	0.69
First 45 d ^5^	45.3 ± 1.4	42.4 ± 1.4	44.3 ± 1.4	43.4 ± 1.4	0.15	0.63
Last 45 d ^6^	31.2 ± 2.9	37.5 ± 2.8	33.7 ± 2.8	35.0 ± 2.8	0.14	0.75

Data presented as LSM ± SEM; ^1^ Age at weaning: early = 150 ± 11 d (n = 24); traditional = 210 ± 11 d (n = 24); ^2^ n is 24 per treatment from d 14 to d 45 and after harvest of 12 animals is reduced to an n of 12; ^3^ d 14 through d 1 of treatment (n = 48); adaption period for the feeding trial; ^4^ Start of treatment; first day of the feeding trial for 45 D and 90 D steers; ^5^ d 0 through d 45 of treatment (n = 48); length of treatment for 45 D steers; first half of the feeding trial for 90 D steers; ^6^ d 46 through d 90 of treatment (n = 24); second half of the feeding trial for 90D steers. There was no significant 2 or 3 way interactions (*p* > 0.37), so main effects are presented.

**Table 2:** Carcass characteristics and proximate analysis values of young steers supplemented with a control (CON) or rumen by-pass (RBL) treatment for either 45 or 90 d (days).

Treatment	CON		RBL		Weaning		*p*-Value			
Days on Feed	45	90	45	90	Early	Traditional	Weaning	Treatment	Days	Treatment × Day
n	12	12	12	12	24	24				
Carcass Measurements										
Hot carcass weight, Kg	130 ± 4	154 ± 4	135 ± 4	153 ± 4	132 ± 3	155 ± 3	<0.001	0.65	<0.0001	0.50
Dressing %	48 ± 1	51 ± 1	49 ± 1	50 ± 1	49 ± 1	49 ± 1	0.77	0.79	0.01	0.27
Ribeye area, cm^2^	19.8 ± 0.8	21.3 ± 0.8	19.6 ± 0.8	20.1 ± 0.8	20.1 ± 0.5	20.2 ± 0.5	0.98	0.27	0.19	0.46
Marbling Score ^1^	174 ± 15	250 ± 15	262 ± 15	306 ± 15	255 ± 11	242 ± 11	0.41	<0.0001	0.0002	0.29
Proximate Analysis										
Dry Matter %	24.5 ± 0.3	25.4 ± 0.3	25.3 ± 0.3	24.6 ± 0.3	24.9 ± 0.3	25.0 ± 0.3	0.70	0.96	0.73	0.02
Ether extract, %	2.1 ± 0.3	2.7 ± 0.3	3.3 ± 0.3	4.0 ± 0.3	3.1 ± 0.2	2.9 ± 0.2	0.73	<0.0001	0.02	0.72

Data presented as LSM ± SEM. ^1^ Marbling score: 100 = practically devoid 00; 200 = devoid 00; 300 = slight 00. There were no additional 2 or 3 way interactions that were significant (*p* > 0.53).

**Table 3:** Specific and total serum fatty acids (mg/mL) on d 0 of steers that were early weaned at 150 d of age (n = 24) or traditionally weaned at 210 d of age (n = 24) and then supplemented with a rumen by-pass lipid ration (RBL) or an isocaloric, isonitrogenous control diet (CON).

Fatty Acid	Early Wean		Traditional Wean			*p*-Value	
	CON	RBL	CON	RBL	SEM	Weaning	Treatment
12:0	4.07	3.94	4.38	3.49	0.35	0.85	0.16
14:0	4.89	4.25	5.90	5.14	0.42	0.03	0.10
16:0	111.07	101.95	118.17	106.59	8.72	0.51	0.50
16:1	18.74	16.88	18.83	17.60	1.90	0.83	0.42
18:0	176.75	158.56	197.05	178.09	16.39	0.23	0.26
18:1 cis 9	12.52	7.33	5.37	4.48	2.44	0.05	0.22
18:1 tran 9	112.95	94.33	119.04	114.77	9.49	0.17	0.23
18:2	277.30	233.42	235.97	186.30	21.22	0.04	0.03
20:0	8.50	9.45	11.62	12.05	1.25	0.03	0.58
18:3	27.76	24.05	37.92	29.20	3.67	0.04	0.10
C20:4	27.45	23.70	25.67	23.29	2.02	0.60	0.14
Total FA	854.28	746.32	848.63	758.8.	66.82	0.95	0.15

There was no significant 2 way interactions (*p* > 0.53) so main effects are presented.

**Table 4:** Specific and total serum fatty acids (mg/mL) on d 45 of steers that were early weaned at 150 d of age (n = 24) or traditionally weaned at 210 d of age (n = 24) and then supplemented with a rumen by-pass lipid ration (RBL) or an isocaloric, isonitrogenous control diet (CON).

Fatty Acid	Early Weaning		Traditional Weaning			*p*-Value		
	CON	RBL	CON	RBL	SEM	Weaning	Treatment	W × T ^1^
12:0	5.38	6.83	5.54	5.99	0.40	0.41	0.02	0.22
14:0	7.75	9.08	7.55	8.32	0.51	0.36	0.05	0.59
16:0	141.8	252.45	144.66	225.10	12.01	0.32	<0.0001	0.22
16:1	23.04	20.35	19.48	15.44	1.45	0.006	0.03	0.64
18:0	216.85	342.48	227.49	314.32	19.94	0.66	<0.0001	0.34
18:1 cis 9	9.69	16.79	6.07	6.62	1.39	<0.0001	0.009	0.02
18:1 tran 9	156.08	150.53	142.42	141.79	11.79	0.35	0.79	0.84
18:2	277.32	694.18	285.18	622.14	33.22	0.34	<0.0001	0.24
20:0	15.12	21.82	11.37	15.70	1.12	<0.0001	<0.0001	0.30
18:3	43.31	49.87	41.21	42.32	3.52	0.18	0.28	0.44
C20:4	31.54	36.85	35.75	37.75	2.34	0.65	0.03	0.95
Total FA	1014.44	1737.40	1043.60	1547.35	90.67	0.38	<0.0001	0.23

^1^ Weaning by RBL treatment interaction.

**Table 5:** Specific and total serum fatty acids (mg/mL) on d 90 of steers that were early weaned at 150 d of age (n = 12) or traditionally weaned at 210 d of age (n = 12) and then supplemented with a rumen by-pass lipid ration (RBL) or an isocaloric, isonitrogenous control diet (CON).

Fatty Acid	Early Wean		Traditional Wean			*p*-Value		
	CON	RB	CON	RB	SEM	Weaning	Treatment	WG ×Trt ^1^
12:0	5.28	5.69	8.16	6.97	0.48	0.0004	0.43	0.12
14:0	7.67	7.86	11.04	9.82	0.61	0.0004	0.42	0.27
16:0	155.97	216.47	223.24	277.61	13.14	0.0004	0.0001	0.82
16:1	20.92	15.52	30.66	20.87	2.25	0.004	0.004	0.34
18:0	252.73	273.29	368.88	411.43	24.97	0.0001	0.20	0.74
18:1 cis 9	8.48	12.57	11.67	10.38	0.97	0.62	0.18	0.01
18:1 tran 9	158.00	131.66	232.97	187.48	12.45	<0.00001	0.01	0.46
18:2	356.74	619.32	432.69	824.93	46.33	0.008	<0.0001	0.19
20:0	17.67	18.04	21.97	20.97	1.74	0.06	0.86	0.70
18:3	37.14	38.12	65.37	59.72	4.08	<0.0001	0.58	0.43
C20:4	37.34	45.23	51.92	60.94	3.74	0.0009	0.04	0.88
Total FA	1156.30	1437.57	1644.00	20638.46	99.04	<0.0001	0.003	0.49

^1^ Weaning (WG) by treatment interaction.

## Supplementary Materials

The following are available online at https://www.mdpi.com/2076-2615/10/10/1819/s1, Table S1: Fatty acid composition of the rumen bypass lipid (RBL), Essentiom, corn gluten feed (CGF), and Bermuda grass hay fed to steers. All items except DM are presented on a DM basis.

## References

[1] Harper G.S., Pethick D.W. (2004). How might marbling begin?. Asian Australas J. Anim. Sci..

[2] Wood J.D., Enser M., Fisher A.V., Nute G.R., Sheard P.R., Richardson R.I., Hughes S.I., Whittington F.M. (2008). Fat deposition, fatty acid composition and meat quality: A review. Meat Sci..

[3] Jost L.K., Dinkel C.A., Costello W.J. (1983). Beef tenderness and palatability as influenced by chemical measures and quality and yield grade factors. JAS.

[4] Rasby R. (2007). Early weaning beef calves. Vet. Clin. N. Am. Food Anim. Pract..

[5] Meyer D.L., Kerley S.M., Walker E.L., Keisler D.H., Pierce V.L., Schmidt T.B., Stahl C.A., Linville M.L., Berg E.P. (2005). Growth rate, body composition, and meat tenderness in early vs. traditionally weaned beef calves. JAS.

[6] Moriel P., Johnson S.E., Vendramini J.M., McCann M.A., Gerrard D.E., Mercadante V.R., Hersom M.J., Arthington J.D. (2014). Effects of calf weaning age and subsequent management systems on growth performance and carcass characteristics of beef steers. JAS.

[7] Myers S.E., Faulkner D.B., Nash T.G., Berger L.L., Parret D.F., McKeith F.K. (1999). Performance and carcass traits of early-weaned steers receiving either a posture growing period or a finishing diet at weaning. JAS.

[8] Scheffler J.M., McCann M.A., Greiner S.P., Jiang H., Hanigan M.D., Bridges G.A., Lake S.L., Gerrard D.E. (2014). Early metabolic imprinting events increase marbling scores in fed cattle. JAS.

[9] Schoonmaker J.P., Lowerch S.C., Fluharty F.L., Zerby H.N., Turner T.B. (2002). Effect of age at feedlot entry on performance and carcass characteristics of bulls and steers. JAS.

[10] Mangrum K.S., Tuttle G., Duckett S.K., Sell G.S., Krehbiel C.R., Long N.M. (2016). The effect of supplementing rumen undegradable unsaturated fatty acids on marbling in early-weaned steers. JAS.

[11] Jenkins T.C. (1993). Lipid metabolism in the rumen. JDS.

[12] Wang Y.H., Bowe N.I., Reverter A., Tan S.H., De Jager N., Wang R., McWilliam S.M., Cafe L.M., Greenwood P.L., Lehnert S.A. (2009). Gene expression patterns during intramuscular fat development in cattle. JAS.

[13] USDA (1997). Official United States Standards for Grades of Carcass Beef. Agricultural Marketing Service.

[14] AOAC (Association of Official Analytical Chemists) (1990). Association of Official Analytical Chemists. https://archive.org/stream/gov.law.aoac.methods.1.1990/aoac.methods.1.1990_djvu.txt.

[15] Long N.M., Burns T.A., Duckett S.K., Schafer D.W. (2014). Reproductive performance and serum fatty acid profiles of underdeveloped beef heifers supplemented with saturated or unsaturated rumen bypass fat compared to an isocaloric control. PAS.

[16] Long N.M., Schafer D.W. (2013). Sex effects on plasma leptin concentrations in newborn and postnatal beef calves. PAS.

[17] Park P.W., Goins R.E. (1994). In situ preparation of fatty acid methyl esters for analysis of fatty acid composition in foods. J. Food Sci..

[18] Long N.M., Tousley C.B., Underwood K.R., Paisley S.I., Means W.J., Hess B.W., Du M., Ford S.P. (2012). Effects of early–to mid–gestational undernutrition with or without protein supplementation on offspring growth, carcass characteristics, and adipocyte size in beef cattle. JAS.

[19] Bolte M.R., Hess B.W., Means W.J., Moss G.E., Rule D.C. (2002). Feeding lambs high-oleate or high-linoleate safflower seeds differentially influences carcass fatty acid composition. JAS.

[20] De Fries C.A., Neundorff D.A., Randel R.D. (1998). Fat supplementation influences postpartum reproductive performance in brahman cows. JAS.

[21] Garcia M.R., Amstalden M., Morrison C.D., Keisler D.H., Williams G.L. (2003). Age at puberty, total fat and conjugated linoleic acid content of carcass, and circulating metabolic hormones in beef heifers fed a diet high in linoleic acid beginning at four months of age. JAS.

[22] Arthington J.D., Spears J.W., Miller D.C. (2005). The effect of early weaning on feedlot performance and measures of stress in beef calves. JAS.

[23] Barker-Neef J.M., Buskirk D.D., Black J.R., Doumit M.E., Rust S.R. (2001). Biological and economic performance of early-weaned angus steers. JAS.

[24] Bruns K.W., Pritchard R.H., Boggs D.I. (2004). The relationships among body weight, body composition, and intramuscular fat content in steers. JAS.

[25] May S.G., Dolezal H.G., Gill D.R., Ray F.K., Buchanan D.S. (1992). Effects of days fed, carcass grade traits, and subcutaneous fat removal on postmortem muscle characteristics and beef palatability. JAS.

[26] Van Kovevering M.T., Gill D.R., Owens F.N., Dolezal H.G., Strasia C.A. (1995). Effect of time on feed on performance of feedlot steers, carcass characteristics, and tenderness and composition of longissimus muscles. JAS.

[27] Greenwood P.L., Siddell J.P., Walmsley B.J., Geesink G.H., Pethick D.W., McPhee M.J. (2015). Postweaning substitution of grazed forage with a high-energy concentrate has variable long-term effects on subcutaneous fat and marbling in Bos taurus genotypes. JAS.

[28] Cooke R.F., Bohnert D.W., Moriel P., Hess B.W., Mills R.R. (2011). Effects of polyunsaturated fatty acid supplementation on ruminal in situ forage degradability, performance, and physiological responses of feeder cattle. JAS.

[29] Hale D.S., Goodson K., Savell J.W., USDA Beef Quality and Yield Grades. https://meat.tamu.edu/beefgrading.

[30] Hocquette J.F., Gondret F., Baéza F., Médale C., Jurie D.W., Pethick D.W. (2010). Intramuscular fat content in meat-producing animals: Development, genetic and nutritional control, and identification of putative markers. Animal.

[31] Laderia M.M., Schoonmaker J.P., Gionbelli M.P., Dias J.C.O., Gionbelli T.R.S., Carvalho J.R.R., Teixaira P.D. (2016). Nutrigenomics and beef quality: A review about lipogenesis. Int. J. Mol. Sci..

[32] Cianzio D.S., Topel D.G., Whitehurst G.B., Beitz D.C., Self H.L. (1985). Adipose tissue growth and cellularity: Changes in bovine adipocyte size and number. JAS.

[33] Hood R.L., Allen C.E. (1973). Cellularity of bovine adipose tissue. J. Lipid. Res..

[34] Azain M.J. (2004). Role of fatty acids in adipocyte growth and development. JAS.

[35] Wang P., Mariman E., Renes J., Keijer J. (2008). The secretory function of adipocytes in the physiology of white adipose tissue. J. Cell. Phys..

[36] Long N.M., Hill G.M., Baker J.F., Graves W.M., Froetschel M.A., Keisler D.H., Mullinix B.G. (2007). Reproductive performance of beef heifers supplemented with corn gluten feed and rumen-protected fat before breeding. PAS.

[37] Hashem N.M., El-Zarkouny S.Z. (2013). Effect of short-term supplementation with rumen-protected fat during the late luteal phase on reproduction and metabolism of ewes. J. Anim. Physiol. Anim. Nutr..

[38] Cartiff S.E., Fellner V., Eisemann J.H. (2013). Eicosapentaenoic and docosahexaenoic acids increase insulin sensitivity in growing steers. JAS.

[39] Cook E.K., Garcia-Ascolani M.E., Ricks R.E., Duckett S.K., Lamb G.C., DiLorenzo N., Long N.M. (2017). The effect of frequency of supplementing rumen-protected unsaturated fatty acids on blood serum fatty acid profiles in beef heifers and lactating cows. JAS.

