# Supplementary material for: The Effects of Age at Weaning and Length of Lipid Supplementation on Growth, Metabolites, and Marbling of Young Steers
Source: Animals (Basel). 2020 Oct 6;10(10):1819. doi: 10.3390/ani10101819 (PMC7600868; doi:10.3390/ani10101819)
Supplement: Supplementary file 1 [file animals-10-01819-s001.pdf]

Supplemental Table 1. Fatty acid composition of the rumen bypass lipid (RBL), Essentiom, corn gluten feed (CGF), and Bermuda grass hay fed to steers. All items except DM are presented on a DM basis

Item	RBL, Essentiom,	CGF	Bermuda hay
DM, %	96.9	91.6	96.4
Ether extract, %	84.5	5.2	2.3
C12:0, %	0.0	0.0	0.0
C14:0, %	0.0	0.0	0.0
C16:0, %	21.8	0.5	0.4
C16:1, %	0.0	0.0	0.0
C18:0, %	3.2	0.1	0.0
C18:1 *t,* %	0.0	0.0	0.0
C18:1 *c,* %	27.7	1.4	0.1
C18:2, %	26.2	3.1	0.4
C18:3, %	4.0	0.1	0.9
Other LCFA[1] , %	1.0	0.0	0.0

[1]Long Chain Fatty Acid (LCFA)
